# A feasible and cost-effective method to induce distal radius fractures in full-length forearm human cadaveric specimens

**DOI:** 10.1007/s00402-026-06549-0

**Published:** 2026-11-03

**Authors:** Julius Krüger, Mads Terndrup, Morten Grove Thomsen, Arvind von Keudell, Jonas Askø Andersen, Tazio Maleitzke

**Affiliations:** 1 Trauma Orthopaedic Research Copenhagen Hvidovre (TORCH), Department of Orthopaedic Surgery, Copenhagen University Hospital – Amager and Hvidovre, Hvidovre, Denmark; 2 Center for Musculoskeletal Surgery, Charité – Universitätsmedizin Berlin, corporate member of Freie Universität Berlin and Humboldt-Universität zu Berlin, Berlin, Germany; 3 Berlin Institute of Health at Charité – Universitätsmedizin Berlin, Julius Wolff Institute, Berlin, Germany; 4 Exo360, Copenhagen, Denmark; 5 Department of Orthopaedic Surgery, Copenhagen University Hospital – Bispebjerg, Copenhagen, Denmark; 6 Department of Clinical Medicine, University of Copenhagen, Copenhagen, Denmark; 7 Department of Orthopaedic Surgery, Brigham and Women’s Hospital, MassGeneral Brigham Harvard Medical School, Boston, USA; 8 Department of Orthopaedic Surgery, Copenhagen University Hospital – North Zealand Hospital, Hillerød, Denmark; 9 Steno Diabetes Center Copenhagen, Copenhagen, Denmark

**Keywords:** Wrist fracture, Biomechanical model, Drop tower, Immobilization device

## Abstract

**Introduction:**

Reported rates of loss of reduction in nonoperatively treated distal radius fractures (DRF) range from 17 to 71%. Thus, realistic biomechanical models are warranted to test novel immobilization devices that prevent loss of reduction in nonoperative fracture management. Currently available human cadaveric specimen models have several limitations: (i) disrupted soft tissue integrity, (ii) difficulty in replicating complex fracture patterns, and (iii) high technical demands and costs. Therefore, the aim of this study was to establish a feasible and accessible method for inducing DRFs, that accurately reflects in-vivo biomechanical fracture conditions in tissue-intact cadaveric specimens, suitable for testing of novel immobilization devices.

**Materials and methods:**

Fifteen upper extremity cadaveric specimens were included. Specimens were prepared as full‑length forearm models. Using a repurposed strength training tower as a vertical drop tower, fractures were induced by proximally administering 120–135 J impacts. CT scans were performed pre- and post-fracture induction. All fractures were classified according to the AO/OTA classification.

**Results:**

DRFs were successfully induced in all 15 specimens. A single 120 J impact produced a DRF in 11 out of 15 cases (73%). A second impact was administered in 4 out of 15 specimens (27%). Eleven of the 15 induced DRFs (73%) were classified as AO/OTA type C fractures, most commonly 2R3C3.2. Concomitant distal ulnar fractures were present in 6 cases, concomitant carpal fractures in 9 specimens. The overall cost of the setup amounted to 947 €.

**Conclusions:**

This study presents a feasible, cost-effective method to induce DRFs in tissue-intact, full-length human cadaveric forearm specimens. The setup consistently produces DRFs, predominantly AO/OTA type C, which can, with certain limitations, be utilized for biomechanical testing of immobilization devices, while also being uited for surgical and anatomical education.

## Introduction

Current nonoperative treatment concepts for distal radius fractures (DRFs) have significant limitations. Reported rates of loss of reduction, ranging from 17 to 71% [1–4], indicate a lack of immobilization devices capable of maintaining fracture alignment over a prolonged period. A loss of fracture reduction may lead to impaired wrist function, malunion or development of secondary osteoarthritis of the wrist [5–7]. Considering that DRFs are among the most common fractures [8, 9], with a majority being treated nonoperatively [10], there is a need for the development of novel immobilization devices that retain fracture reduction over several weeks. Consequently, realistic biomechanical models are warranted to evaluate such devices.

Currently available biomechanical models using human cadaveric specimen fail to provide realistic conditions. Cutting osteotomy models [11, 12] are artificial, disrupt the integrity of the soft tissue surrounding the radius and fail to replicate the complex biomechanical mechanisms of intra-articular or joint near fractures, making them unsuitable for biomechanical testing of immobilization devices.

A method for inducing “life-like” DRFs in human cadaveric specimens, was previously described by Wegmann et al. [13]. The group produced realistic fracture patterns of the distal radius by using high energetic impulses induced with a custom-made drop-test-bench. The setup is technically demanding and cost intensive. Furthermore, the approach is based on short forearm specimens, which are unsuitable for cast or device application to the wrist and subsequent biomechanical testing.

Therefore, the aim of this study was to establish an accessible method for reliably inducing DRFs, that accurately reflects in-vivo biomechanical fracture conditions in tissue-intact, full-length human cadaveric forearm specimens, suitable for biomechanical testing of novel immobilization devices.

## Materials and methods

This study was conducted on fresh‑frozen cadaveric specimens from deceased individuals who had bequeathed their bodies to science and education, according to Danish legislation (health law #546, § 188). Approval was obtained from the head of the Body Donation Programme at the Department of Cellular and Molecular Medicine, University of Copenhagen. Further approval from the Data Protection Agency was not required as no personal information, age or gender of the individuals was known.

### Specimen preparation

Fifteen fresh‑frozen upper extremities with intact soft tissues, severed at mid-humeral level, were included in this study. Using radiography before defrosting, specimens with prior wrist surgery, implants, marked deformity or advanced arthropathy were not included.

**Fig. 1:**
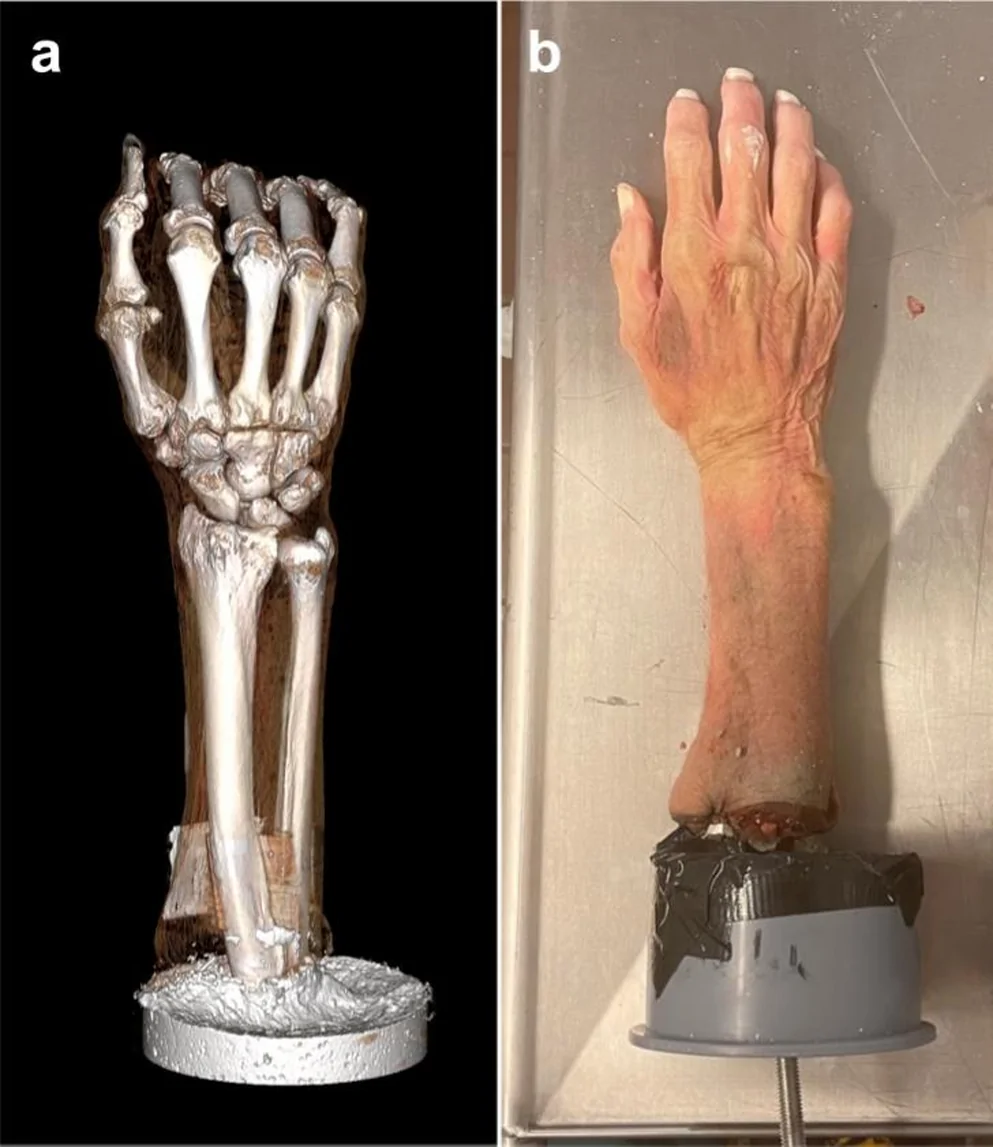
Pre-fracture CT 3D reconstruction scan (**a**) and photograph (**b**) of specimen

The specimens were thawed overnight at room temperature. For proof of concept, specimen I was prepared based on the approach described by Wegmann et al. [13] as a short forearm model. Specimens II–XV were prepared as full‑length forearm models.

Through an anterior approach across the elbow crease, the humeroradial and humeroulnar joint were disarticulated. An olecranon osteotomy was performed at the level of the radial head. The soft tissue was dissected from the bone up to 5 cm distally to the osteotomy. All forearm muscles originating from the distal humerus were transected to expose a proximal bone segment for cement fixation. The soft‑tissue envelope distal to this segment remained intact (Fig. 1a–b).

The proximal bone segments were potted within a polyvinyl chloride (PVC) pipe cap (Wafix afløbsprop, Ø110 mm, Nordisk Wavin A/S, Denmark), using fast drying cement (Skalflex^®^ Lyn-cement, Sika Danmark A/S, Denmark). The specimens were manually supported for 10 min until the cement had hardened and was self-supporting. The total drying time was three hours. A transverse M10 bolt through the center of the bottom of the PVC cap allowed secure fixation in the drop tower.

### Apparatus

**Fig. 2:**
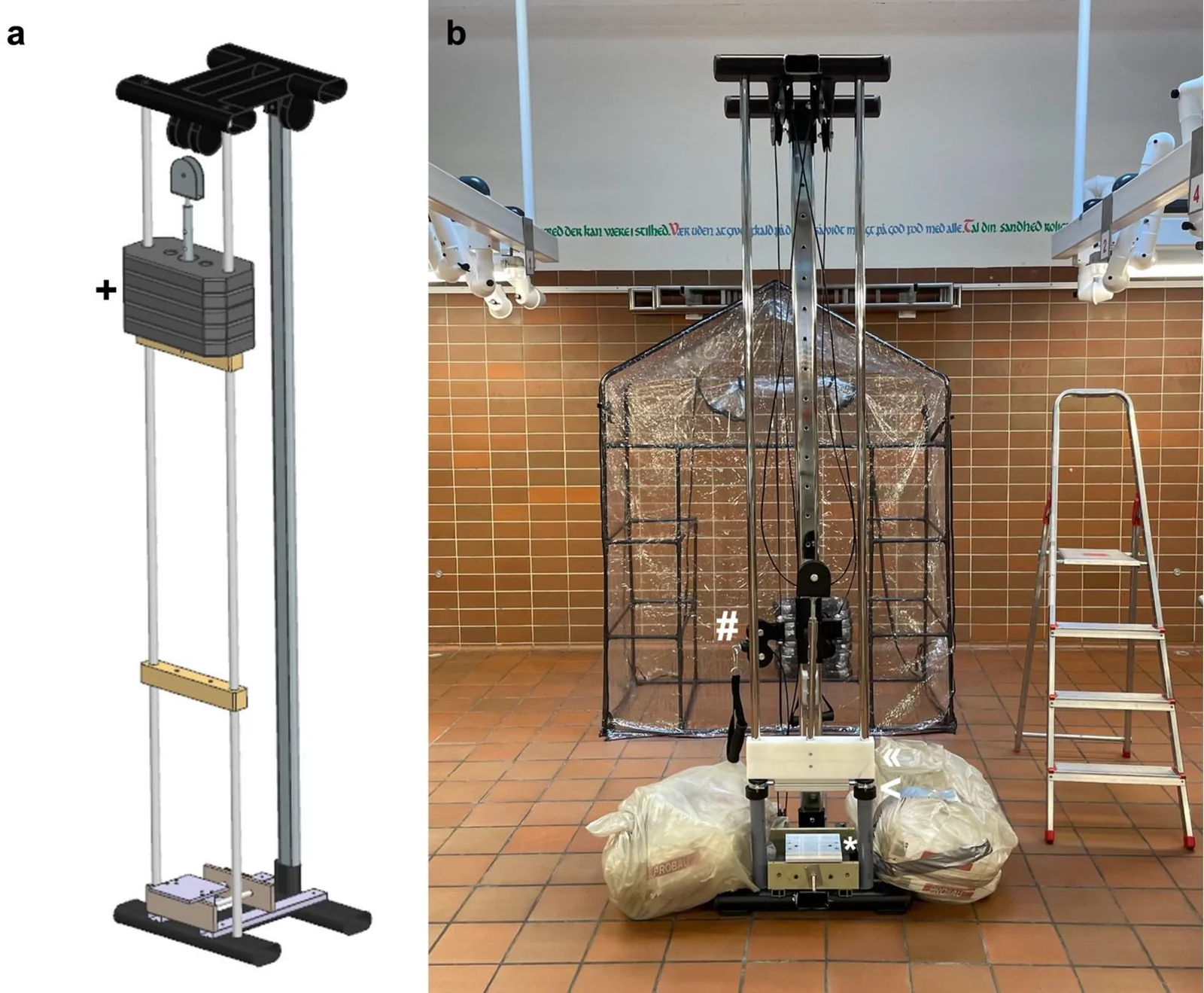
Virtual model (**a**) and setup (**b**) of vertical drop tower including a quick-release mechanism (#), cross beam (< ), polymer block (**«**), base plate (*) and stackable mass plates (+)

A strength training tower (Abilica FlexTower 2.0, Mylna Sport AS, Norway) was repurposed as a vertical drop tower (Fig. 2a–b) by: (i) removing the wire wheel and welding it to the bottom of the tower, creating a quick-release mechanism (Fig. 2b#), (ii) adding an aluminum cross beam with tapped holes (Fig. 2b< ) for bolt mounting, (iii) adding a polymer block (Fig. 2b«), and (iv) adding a base plate (Fig. 2b*). The guided carriage accommodated stackable mass plates (Fig. 2a+) with indexed height settings, ensuring a reproducible drop mass and height. Safety measures included a polycarbonate shield, floor stops, and a setup tether.

### Fracture induction and impact protocol

**Fig. 3:**
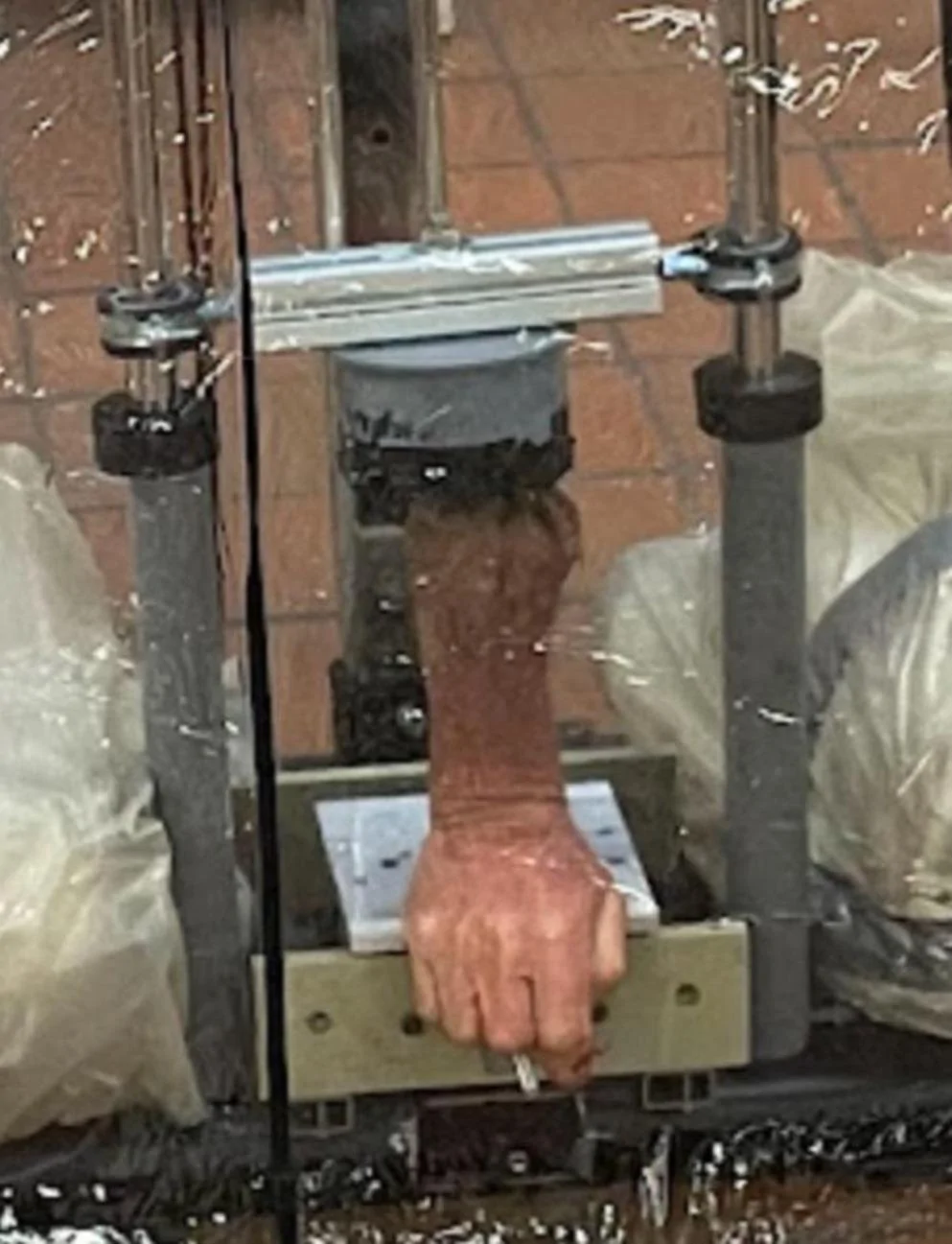
Placement of specimen in the vertical drop tower

The specimens were positioned with approximately 80° of dorsal extension in the wrist and a neutral position of the forearm, ensuring the axial impact is applied centrally (Fig. 3).

Impacts of 120 J (calculated as E = mgh) were induced by releasing a fixed moving mass of 13.8 kg from a height of 89 cm. If a fracture was absent after the first impact, a second impact was permitted with up to 135 J. Wrist positioning was not changed when delivering a second impact. Fracture induction was recorded with a camera.

### Imaging and classification

Pre-fracture CT imaging (Siemens Healthineers SOMATOM Definition AS) was performed as a baseline reference and to assess bone structure and integrity. Mean Hounsfield units (HU) were calculated for each specimen, as described by Wagner et al. [14], by measuring a circular region of interest (ROI) in the distal ulna at level of the distal radial-ulnar joint without including cortical bone, on three sequential coronal CT slices. Post-fracture CT imaging was conducted to confirm and characterize fracture morphology. All fractures were independently classified according to the AO/OTA classification [15] by two experienced orthopedic trauma surgeons. Discrepancies were resolved by discussion between the two assessors until consensus was reached.

## Results

Nine of the included specimens (60%) were right upper extremities and 6 (40%) were left. Mean HU for all specimens was 195,4 ± 78,8.

DRFs could be induced in all 15 specimens. A single 120 J impact produced a DRF in 11 out of 15 cases (73%). A second impact (with a maximum of 135 J) was administered in 4 specimens (specimens IV, IX, XIII, XV) (27%). In 1 specimen (specimen XIV) the impact resulted in an open fracture.

**Fig. 4:**
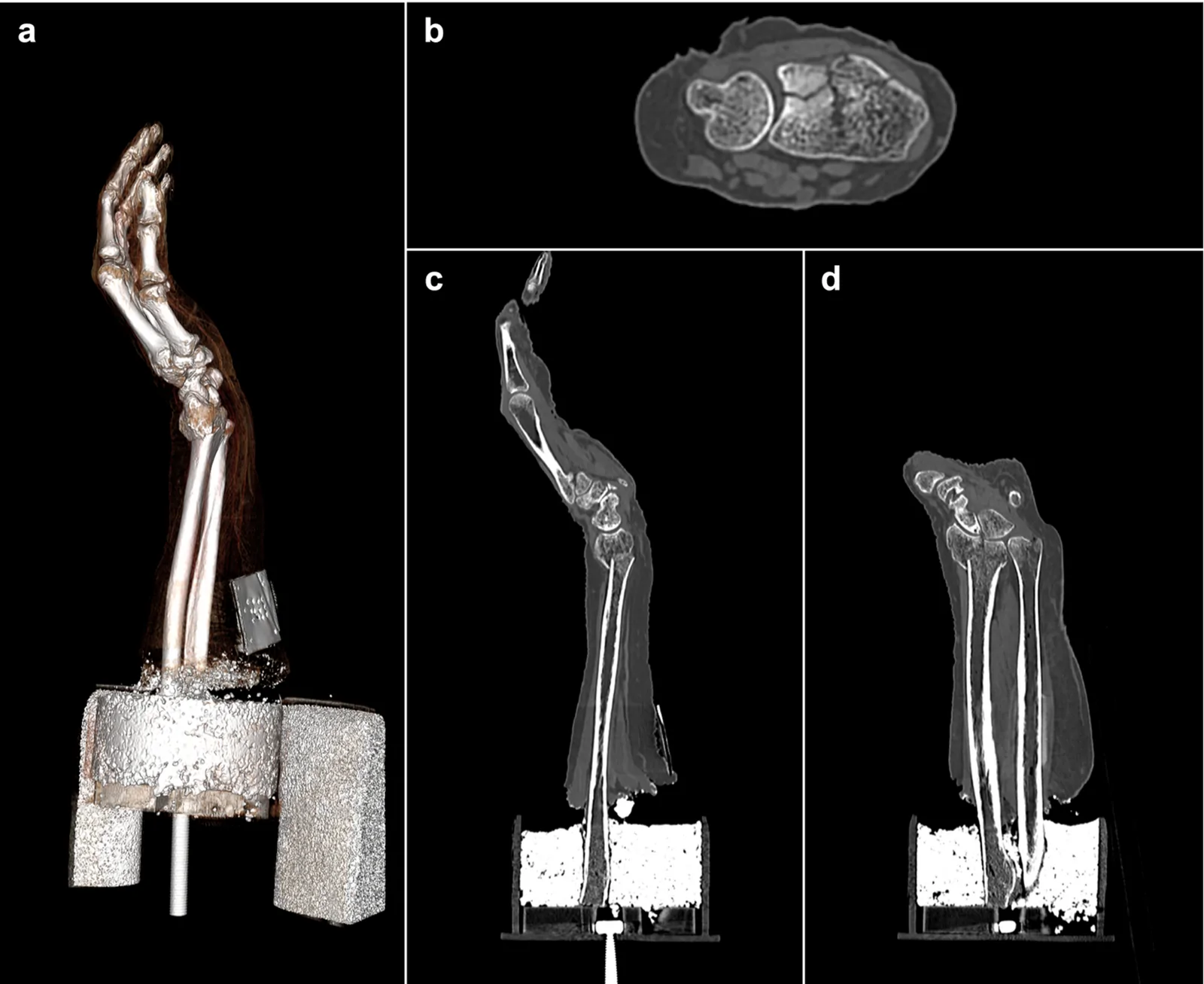
Post-fracture CT scan of specimen XII. 3D reconstruction (**a**), axial (**b**), sagittal (**c**) and coronal (**d**) plane

**Table 1:** Characteristics and induced fractures for each specimen

Specimen	Laterality	Mean HU	Kinetic energy [J]	DRF (AO/OTA)	Displacement of DRF	Ulnar fracture (AO/OTA)	Other concomitant fractures / injuries (AO/OTA)
I	R	84,4	120	C3.1	Dorsal	2U3A3	-
II	R	240,9	120	C3.1	Dorsal	2U3A1.1	Pisiform fracture (76.1.B)
III	R	212,9	120	C3.2	Dorsal	-	-
IV	R	193,2	120, 120	B2.2	Undisplaced	-	Trans-scaphoid perilunate fracture dislocation
V	R	154,4	120	C2.1	Undisplaced	-	-
VI	L	217,0	120	C3.2	Dorsal	2U3A3	Scaphoid fracture (72Bb)
VII	R	163,0	120	C3.2	Dorsal	-	-
VIII	L	375,8	120	B2.1	Undisplaced	2U3A1.1	Scaphoid fracture (72Bb), lunate fracture (71 A), capitate fracture (73 C)
IX	L	310,4	120, 120	C3.2	Dorsal	-	Scaphoid fracture (72Ba)
X	L	165,2	120	B1.3	Dorsal	-	Scaphoid fracture (72Bb)
XI	R	132,0	120	C1.1	Dorsal	2U3A1.1	Scaphoid fracture (72Bb)
XII	L	73,4	120	C3.2	Dorsal	2U3A1.1	-
XIII	R	218,9	120, 135	C3.2	Dorsal	-	-
XIV	L	232,9	120	C1.2	Dorsal	-	Trans-scaphoid perilunate fracture dislocation, trapezium fracture (75B)
XV	R	155,8	120, 120	B2.2	Undisplaced	-	Trans-scaphoid perilunate fracture dislocation, capitate fracture (73 C)

Eleven induced DRFs (73%) were classified as type C fractures according to the AO/OTA Classification [15], most commonly 2R3C3.2. Dorsal displacement was present in 11 DRFs (73%). A representative fracture pattern is presented in Fig. 4a–d. A concomitant distal ulnar fracture was present in 6 specimens. Further, concomitant carpal fractures were seen in 9 specimens, including 8 scaphoid fractures, 2 capitate fractures, 1 lunate fracture, 1 trapezium fracture, and 1 pisiform fracture. Three of the 8 scaphoid fractures were associated with a trans-scaphoid perilunate fracture dislocation. There was an independent 100% agreement between the two surgeons classifying the fractures. All data are summarized in Table 1.

The overall cost of the setup amounted to 947 €. A comprehensive breakdown of the associated costs is presented in Table 2. The breakdown does not include personnel costs for specimen preparation, imaging and workshop fabrication. A total of 15 working hours were spent on building the apparatus. The experiment was performed over a period of two days by six people, and included setup of the drop tower, specimen preparation, fracture induction and CT imaging.

**Table 2:** Cost calculation. Costs are cited as list prices without value-added tax

Item	Quantity	Total amount [€]
Strength training tower	1	292
Steel base stamp	1	299
Aluminum rod	1	70
POM polymer block	1	199
Wavin Wafix drain cap	15	61
M10 bolt	15	14
Skalflex^®^ lyn-cement 5 kg	1	12
All		947

## Discussion

In this study, we present a technically accessible and cost-effective method to consistently generate DRFs in tissue-intact, full-length human cadaveric forearm specimens by using a repurposed strength training tower.

The most common trauma mechanism for DRFs is falling from own height [16–18]. To further reproduce the trauma mechanism of a fall on the outstretched hand in an experimental setup, the specimens were positioned with approximately 80° of dorsal wrist extension and axial impacts were applied centrally to the proximal forearm, generating a bending force over the distal radius. This study setup resulted in successfully induced DRFs in all 15 specimens.

Our methods build upon prior work of Wegmann et al. [13], who demonstrated that high-energy impacts applied to short forearm cadaveric specimens can induce “life-like” DRFs. We first reproduced the approach in a short forearm specimen (specimen I), confirming feasibility of the reference method and subsequently applied it to full-length forearm specimens (specimens II–XV). By using a mean weight of 16.3 kg and a mean kinetic energy of 134.7 J, Wegmann et al. [13] mostly produced AO/OTA type C3.2 DRFs (11 out of 12). Using the same degree of dorsal extension, but a reduced impact weight and target kinetic energy, our study setup has likewise shown to mostly generate AO/OTA type C3.2 DRFs, whilst also inducing AO/OTA type B1, B2 and C1 DRFs. Comparing the mean HU, calculated based on the same measurement technique as Wegmann et al. [13], the specimens utilized in our study had a higher mean HU, indicating a higher bone mineral density [14]. This may have contributed to the differences in fracture comminution and first impact success rate. Specimen XIII was the first specimen in which the initial 120 J impact failed to induce a fracture. For the second impact, the kinetic energy was therefore increased to 135 J, resembling the mean kinetic energy used in the reference method [13]. This resulted in an AO/OTA C3.2 DRF. To minimize the risk of excessive fracture comminution, the impact was subsequently repeated at 120 J rather than immediately escalating to 135 J. This approach successfully induced DRFs in all three other specimens requiring a second impact, without the need for further escalation to 135 J. A similar incidence of concomitant distal ulnar fractures can be observed in both studies. Although not specifically reported by Wegmann et al. [13], a lunate fracture can be seen in the published CT imaging, indicating a possibly similar spectrum and incidence of concomitant carpal fractures.

A major strength of this study setup is its accessibility. The apparatus is based on a commercially available strength training tower and required only basic modifications that can be fabricated using standard workshop tools and off-the-shelf materials. Further, fast drying cement can be used instead of the more cost-intensive polymethyl methacrylate (PMMA). Consequently, the overall cost of the setup could be reduced to 947 €. As such, the setup can be replicated in most cadaver laboratories without the need for specialized or cost-intensive equipment.

The use of tissue-intact, full-length specimens represents an additional key aspect of this method. Fracture models using cutting osteotomy to induce DRFs [11, 12] disrupt the integrity of the soft tissue surrounding the radius. The method by Wegmann et al. [13] produced fractures with intact skin- and soft tissue envelope. To ensure force application to the central aspect of the distal radius and to utilize the proximal bone and elbow joint for further scientific work, the authors chose short forearm cadaveric specimen. This makes both models unsuitable for biomechanical testing of immobilization devices, since they are not allowing cast or device application to the wrist. Here, we can report that the tissue-intact, full-length specimens produced by our study setup were successfully utilized in biomechanical testing of new immobilization devices.

This study has limitations. It must be noted that to confirm feasibility of the reference method [13], specimen I was prepared as a short-forearm specimen, before continuing with full-length specimens. Therefore, only specimens II–XV were prepared and fractured as full-length specimens.

The impact energy was calculated using drop mass and height. While calculated impact energy, and impact counts were recorded, the study setup was not instrumented with force or acceleration sensors. Thus, the actual force transmitted to the specimen might have varied due to energy absorbed by the experimental setup or deviations of impact weight acceleration and contact time.

Further, only one tower configuration and wrist position were evaluated. Due to its complexity and the absence of active muscle tension and protective responses, the trauma mechanism of a fall can in a cadaveric experimental setup only be reproduced in a simplified matter. In contrast to a fall on the outstretched hand, where the force is transmitted through the palm of the hand to the distal forearm, the current study setup, as well as the reference method by Wegmann et al. [13], relies on an anterograde force transmission from the proximal forearm to the wrist. A setup utilizing retrograde force transmission would resemble the trauma mechanism of a fall on an outstretched hand more closely but appears technically challenging and may not be feasible in practice.

The current study setup demonstrated consistent fracture induction, with a 73% success rate following a first impact and a 100% success rate following a second impact when the first impact failed. To further improve method standardization and first impact success rate, future studies may adapt our method and systematically modulate impact energy and degree of dorsal extension of the wrist, potentially inducing reproducible extraarticular (AO/OTA type A) or partially articular (AO/OTA type B) DRFs. Based on our findings, a reduced impact energy might result in a lower degree of comminution and a higher incidence of AO/OTA type B DRFs, whilst potentially decreasing the success rate at first impact and increasing the risk for failure of fracture induction.

Since the increased impact energy in specimen XIII did not result in concomitant carpal fractures or dislocations, the degree of dorsal angulation may be a more relevant factor for concomitant carpal injuries. Reducing dorsal extension of the wrist was not feasible, as it may lead to the specimen slipping during proximal impact application. Pechlaner et al. [19] induced DRFs in cadaveric specimen through hyperextension trauma and constitute the absence of physiological reflexes as an additional relevant factor for concomitant carpal ligament injuries.

Other published studies utilizing test-benches for inducing realistic fractures in cadaveric specimens [20–23] have reported similar challenges in creating a homogenous fracture spectrum. Specimen positioning and location of impact application, as well as individual anatomy of each specimen, appear to influence the induced fracture patterns. In addition, whilst creating realistic fracture patterns, test-bench models seem to induce comminuted fractures with a high incidence of concomitant injuries.

The intended use of our method was to generate a cost-effective and accessible device to induce DRFs to later test new immobilization devices. Most fractures generated by the study setup were AO/OTA type C fractures. Although AO/OTA type C DRFs are, especially in patients with high functional demands, frequently treated surgically [16, 24], a relevant proportion are treated nonoperatively [18]. Therefore, biomechanical evaluation of fracture stability and displacement under mechanical stress in DRFs is feasible. Nonetheless, the fracture complexity and the absence of active muscle tension must be considered when interpreting the results. Novel immobilization devices should therefore be evaluated in direct comparison with a control device group in future biomechanical studies. As post-fracture swelling does not occur in cadaveric specimens, the model has limitations when assessing secondary loss of reduction, development of pressure injuries or compartment syndrome. In practice, patients with trans-scaphoid perilunate fracture-dislocations would rarely be suited for nonoperative treatment.

Therefore, we suggest that the fracture spectrum generated by our model can additionally be used for anatomical education and surgical training. The intact soft-tissue envelope allows training of surgical approaches and fixation techniques.

## Conclusion

This study presents and establishes a feasible, cost-effective methodology for inducing DRFs in tissue-intact, full-length human cadaveric forearm specimens by repurposing a strength training tower into a vertical drop tower. The setup consistently produced DRFs, predominantly AO/OTA type C, with concomitant distal ulnar fractures and some associated carpal injuries. The fracture model can, with certain limitations, be utilized for biomechanical testing of immobilization devices, while being well suited for surgical and anatomical education.

## Data Availability

The data generated and analysed during the current study are available from the corresponding author on reasonable request.
